# Health Tract, No. 289

**Published:** 1867-05

**Authors:** 


### CONTRADICTIONS.

If a man were to say to me that the moon was made out of a monkey, I would say nothing, and let him have his own way; either first, because "he was a fool and had no sense," as our mother used to say in an expressive manner peculiar to herself; or second, that he knew better and wanted to provoke argument, and I have seldom found argument upon any subject, especially on politics or religion, either profitable or agreeable; this is particularly the case, if any third person is present; for then, the contest is for victory, and not truth; each one is ashamed to be vanquished, and has an ambition to be appreciated as the better or wiser man, by those who are present, and in the heat of the contest, each closes his eyes to the force of the other's arguments, and makes exaggerated statements, if not downright fabrications, for the support of his own views. If your antagonist is unintelligent, he has a double advantage over you; he is too obtuse to see the force of your argument, or so self-opinionated that he will not see it; besides, when hard pushed, he will make some statement to help him out of the scrape, that is so palpably absurd, that it puts you in a rage, and that moment you make a fool of yourself, and there are two donkeys, instead of one; your equanimity is departed, your "bile has been stirred up," literally, and you "feel bad," and are bad in temper and bodily condition for hours together; and all this, because a numskull had said to you that the moon was made out of a monkey; a very

"Ocean into tempest tost,

To waft a feather, or to drown a fly."

O ye married folk, all over creation, how much of the nectar of domestic bliss becomes "spilt milk"! because of contradictions upon matters too trivial for a second thought. If I make a statement at the table that it rained yesterday at one o'clock, and my wife says, "No my dear, you are mistaken, for the clock struck one, just as we got inside the door, and it was at least a minute before the drops began to fall," now why should I reply to that? Suppose it began to rain a minute sooner or later, it is of no consequence as to the general statement that "it rained yesterday at one o'clock," and yet, in this way, millions of domestic quarrels have commenced, ending in unhappy estrangements for hours, months, years!! Reader, never contradict, especially your wife, for she will always get the advantage sooner or later. Let your replies be deprecatory. If she says "you are a fool," don't argue the case; and if you say anything at all let it be, "that's so," but don't add, "else I would never have married you."

